# Prognostic value of metabolic tumor volume of extranodal involvement in diffuse large B cell lymphoma

**DOI:** 10.1007/s00277-023-05165-x

**Published:** 2023-03-23

**Authors:** Kana Oiwa, Kei Fujita, Shin Lee, Tetsuji Morishita, Tetsuya Tsujikawa, Eiju Negoro, Takeshi Hara, Hisashi Tsurumi, Takanori Ueda, Takahiro Yamauchi

**Affiliations:** 1 Department of Hematology and Oncology, Faculty of Medical Sciences, University of Fukui, Fukui, Japan; 2 Department of Hematology and Oncology, Nagoya City University, Aichi, Japan; 3 Department of Hematology and Oncology, Matsunami General Hospital, Dendai 185-1 Kasamatsu-Cho, Hashima-Gun, Gifu, 501-6062 Japan; 4 Department of Internal Medicine, Matsunami General Hospital, Gifu, Japan; 5 Department of Healthcare Economics and Quality Management, Graduate School of Medicine, Kyoto University, Yoshida Konoe-Cho, Kyoto, Japan; 6 Department of Radiology, Faculty of Medical Sciences, University of Fukui, Fukui, Japan; 7 Department of Cancer Care Promotion Center, University of Fukui, Fukui, Japan

**Keywords:** Extranodal involvement, Metabolic tumor burden, Positron emission tomography, Prognosis, Diffuse large B cell lymphoma

## Abstract

Extranodal involvement predicts poor outcomes of diffuse large B cell lymphoma (DLBCL), but the impact of the metabolic tumor burden (MTV) of extranodal sites using positron emission tomography has not been clarified. This study aimed to assess the impact of extranodal MTV on overall survival (OS). We retrospectively analyzed 145 newly diagnosed DLBCL patients and verified the prognostic impact of each extranodal and nodal MTV. Multivariate Cox hazards modelling using both extranodal and nodal MTV as covariables identified extranodal MTV as a significant factor for OS (hazard ratio [HR] 1.072, 95% confidence interval [CI] 1.019–1.129, *P* = 0.008), but not nodal MTV. Multivariate Cox modelling using restricted cubic splines demonstrated that the impact of total MTV depends on the MTV of extranodal sites, not of nodal sites. When both the number and MTV of extranodal involvements were used as covariables, extranodal MTV remained a significant predictor of OS (HR 1.070, 95%CI 1.017–1.127, *P* = 0.009), but the number of extranodal sites did not. Extranodal MTV potentially had a more significant role on prognosis than nodal MTV. When considering prognostic impacts, the MTV of extranodal involvement is significantly more important than the number.

**Supplementary Information:**

The online version contains supplementary material available at 10.1007/s00277-023-05165-x.

## Introduction

Diffuse large B cell lymphoma (DLBCL) is the most common subtype of lymphoma, and is categorized as aggressive [1, 2]. Given the high chemosensitivity and potential curability of the disease, providing optimal treatment for each individual patient is indispensable [3, 4]. The heterogeneity of DLBCL has been identified both clinically and molecularly [5]. DLBCL with an extranodal presentation is genetically and clinically different from nodal DLBCL, showing a negative influence on prognosis [6–10]. The presence of bulky disease or elevated levels of lactate dehydrogenase (LDH) represent a high tumor burden and are also associated with adverse outcomes [11–13].

Positron emission tomography (PET) with 2-[^18^F]fluoro-2-deoxy-D-glucose (FDG) plays an important role in the evaluation of baseline extranodal DLBCL [14, 15]. Metabolic tumor volume (MTV) measured by FDG-PET is a parameter reflecting the tumor burden, taking into account the metabolic activity of the tumor. Previous studies have demonstrated that pretreatment total MTV affects survival outcomes in DLBCL [16–18]. Recently, a new prognostic indicator using MTV, age, and stage has been shown to outperform the prognostic ability of the IPI (International Prognostic Index), leading to greater attention being paid to the prognostic utility of the MTV in DLBCL [19]. However, the prognostic impact of MTV in DLBCL can be expected to differ widely depending on whether extranodal or nodal disease is present. Few reports have focused on the MTV of extranodal involvement in the pretreatment assessment of DLBCL patients, despite its likely importance.

The present study investigated the impact of the baseline MTV of extranodal involvement as measured by FDG-PET on overall survival (OS) in patients with de novo DLBCL using multivariate Cox hazards modelling with restricted cubic splines (RCS). Furthermore, the prognostic impacts of MTV of nodal and total involvement sites were evaluated using Cox hazards modelling.

## Methods

### Patient selection

We performed a retrospective, single-centre observational study from 2007 to 2017 at University of Fukui Hospital, Japan. Lymphomas were classified according to the Revised European American Lymphoma classification and the World Health Organization classification [2, 20]. Patients comprised individuals with newly diagnosed, histologically confirmed DLBCL, who were ≥ 18 years old at diagnosis and who underwent FDG-PET before treatment. Patients with post-transplant lymphoproliferative disorder, central nervous system involvement, composite disease consisting of DLBCL plus indolent non-Hodgkin’s lymphoma, human immunodeficiency virus or unassessable FDG-PET due to hyperglycaemia were excluded. To avoid bias when assessing the survival outcome, the primary transformation from indolent lymphoma was excluded in the present study. Patients who had most tumors surgically removed before the initiation of chemotherapy were also excluded.

The baseline demographics and disease characteristics of patients were collected by retrospective chart review. Baseline characteristics including Eastern Cooperative Oncology Group performance status (PS), number of extranodal sites, elevated LDH level (> 222 IU/L), soluble interleukin-2 receptor (sIL-2R) level, serum albumin level, bulky mass (maximum diameter > 7.5 cm), B symptoms, bone marrow involvement, and the IPI were extracted. Comorbidities at diagnosis were assessed using the Charlson Comorbidity Index (CCI) [21].

### FDG-PET and measurement of MTV

All whole-body PET scans with FDG were performed using a combined PET/CT scanner (Discovery LS; GE Medical Systems), which permits simultaneous acquisition of 35 image slices in 3-dimensional acquisition mode with inter-slice spacing of 4.25 mm. The PET/CT scanner incorporates an integrated four-slice multidetector CT scanner, which was used for attenuation correction. CT scanning parameters were as follows: Auto mA (upper limit, 40 mA; noise index, 20); 140 kV; section thickness, 5 mm; table feed, 15 mm; and pitch, 4 mm. After fasting for at least 4 h, patients received intravenous injection of 185 MBq of FDG and image acquisition began 50 min after injection. A whole-body emission scan was performed from the head to the inguinal region, with 2 min per bed position (7–8 bed positions). PET data were reconstructed by the iterative reconstruction method selecting 14 subsets and 2 iterations. Reconstructed images were then converted to a semi-quantitative image corrected by injection dose and body weight of the subject (= standardized uptake value [SUV]).

Total MTV was defined as the sum of the metabolic volumes of all lymphoma involvements with SUV greater than or equal to an absolute threshed of 4.0, as previously reported [22–25]. Nodal MTV was defined as the sum of the MTVs of all nodal involvements and extranodal MTV was defined as the sum of the MTVs of all extranodal involvements as measured by FDG-PET. MTV was measured using the Metavol software (Hokkaido University, Sapporo, Japan; http://www.metavol.org). The radiologists with expertise in reading FDG-PET images evaluated all accumulation sites, including nodal and extranodal involvements, on FDG-PET in all cases. Based on these evaluation reports, nodal extranodal lesions were manually measured using the Metavol software, respectively.

### Outcome measures

The primary outcome was OS. We also assessed prognosticators considered to influence survival outcomes in DLBCL patients. OS was calculated from the date of diagnosis to the date of death due to any cause or the latest follow-up visit. The dates of events were retrospectively collected for all patients by chart review and censored as of September 31, 2021.

### Statistical analysis

Continuous variables are presented as median values with ranges, with groups compared using the Mann–Whitney *U* test. Categorical variables are presented as numbers and percentages, and groups were compared by the chi-squared test or Fisher’s exact test, as appropriate. Cox proportional hazards modelling was applied to calculate hazard ratios (HRs) and 95% confidence intervals (CIs). Multivariate Cox proportional hazards modelling was used to determine prognosticators for all-cause mortality risk. Covariables including sex, age, PS, stage, CCI, total MTV, extranodal MTV, and nodal MTV were used in multivariate analysis. To estimate the impact of extranodal MTV on survival independent of total MTV, we created two different multivariate models: a model with total MTV as a covariable; and a model with extranodal and nodal MTV as covariables. Nonlinear regression model using RCS with 3 knots was used to evaluate the presence of a nonlinear association between MTV and all-cause mortality risk [26]. All *P*-values in the present study were two-sided, with values of *P* < 0.05 considered significant. Data analysis was performed using R (version 4.1.1) or EZR (version 1.55), which is a graphical user interface for R [27, 28].

## Results

### Patient characteristics

A total of 163 patients were identified, then 18 patients who met the exclusion criteria were excluded. The remaining 145 analyzable patients were included in the present study (Supplementary Fig. 1). Table 1 presents patient characteristics at diagnosis for cases with and without extranodal MTV. Median age at baseline was 72 years (range, 27–96 years), and 115 patients (79.3%) had extranodal lesions with measurable MTV. Patients with any measurable extranodal MTV displayed significantly more advanced-stage disease, higher IPI scores, more frequent bone marrow involvement, higher SUV max, and higher total and nodal MTV.

**Table 1:** Patient characteristics at diagnosis

	All patients (*n* = 145)		With FDG accumulation in extranodal lesions (*n* = 115)		Without FDG accumulation in extranodal lesions (*n* = 30)		*P* value
Age, years—median, range	72	(27–95)	72	(27–92)	73	(47–95)	0.938
Male sex—*n* (%)	73	(50.3)	55	(47.8)	18	(60.0)	0.306
ECOG PS ≥ 2—*n* (%)	39	(26.9)	30	(26.1)	9	(30.0)	0.651
Extranodal sites ≥ 2—*n* (%)	60	(41.4)	53	(46.1)	7	(23.3)	0.036
Ann Arbor Stage III/IV—*n* (%)	94	(64.8)	85	(73.9)	9	(30.0)	< 0.001
Elevated LDH (> ULN)—*n* (%)	91	(62.8)	76	(66.1)	15	(50.0)	0.137
Serum albumin, g/dl—median, range	3.5	(1.7–4.7)	3.4	(2.0–4.7)	3.7	(1.7–4.7)	0.101
sIL-2R, U/mL—median, range	1,452	(168–29,970)	1,461	(168–29,970)	1,238	(239–14,871)	0.445
IPI—*n* (%)							
Low (0, 1)	33	(22.8)	25	(21.7)	8	(26.7)	
Low intermediate (2)	30	(20.7)	16	(13.9)	14	(46.7)	< 0.001
High intermediate (3)	30	(20.7)	25	(21.7)	5	(16.7)	
High (4, 5)	52	(35.8)	49	(42.6)	3	(10.0)	
Bulky mass—*n* (%)	30	(20.7)	25	(21.7)	5	(16.7)	0.622
B symptoms—*n* (%)	44	(30.3)	36	(31.3)	8	(26.7)	0.823
Bone marrow involvement—*n* (%)	24	(16.6)	24	(20.9)	0	(0.0)	0.004
CCI—*n* (%)							
0	56	(38.6)	42	(36.5)	14	(46.7)	
1, 2	64	(44.2)	49	(42.6)	15	(50.0)	0.099
3, 4	16	(11.0)	16	(13.9)	0	(0.0)	
≥ 5	9	(6.2)	8	(7.0)	1	(3.3)	
G8 score—median, range	12	(2–17)	12	(2–17)	11	(3–17)	0.544
SUV_max_—median, range	27.1	(4.2–213.6)	28.0	(4.2–213.6)	22.0	(5.0–46.9)	0.021
SUV_max_ of nodal sites—median, range	22.0	(0.0–213.6)	23.4	(0.0–213.6)	21.9	(5.0–46.9)	0.920
SUV_max_ of extranodal sites—median, range	20.4	(0.0–157.4)	26.2	(4.2–157.4)	0.0	(0.0–0.0)	< 0.001
TMTV, cm^3^—median, range	167.5	(0.5–2,515.6)	212.3	(0.5–2,515.6)	53.9	(2.8–876.3)	0.014
MTV of nodal sites, cm^3^—median, range	27.0	(0.0–1,472.5)	15.8	(0.0–1,472.5)	53.9	(2.8–876.3)	0.002
MTV of extranodal sites, cm^3^—median, range	52.1	(0.0–2,459.1)	99.3	(0.5–2,459.1)	0.0	(0.0–0.0)	< 0.001

*CCI*, Charlson Comorbidity Index; *ECOG PS*, Eastern Cooperative Oncology Group performance status; *G8*, Geriatric 8; *IPI*, International Prognostic Index; *LDH*, lactate dehydrogenase; *MTV*, metabolic tumor volume; *SUV*, standardized uptake value; *sIL-2R*, soluble interleukin-2 receptor; *TMTV*, total metabolic tumor volume; *ULN*, upper limit of normal

### Predictors of OS

Median duration of follow-up was 49.6 months (range, 0.03–166.9 months), during which time 65 patients died (44.8%), including 34 deaths (23.4%) due to lymphoma. The 2-year estimated OS in all eligible patients was 73.8%. Multivariate Cox proportional hazard modelling for all-cause mortality using total MTV as a covariable is shown in Table 2. Total MTV (HR 1.059, 95%CI 1.007–1.113, *P* = 0.025) was an independent predictor of OS. To evaluate the prognostic impact of extranodal MTV, we created another multivariate Cox proportional hazard model for all-cause mortality using nodal MTV and extranodal MTV as the covariables (Table 3). The results showed that extranodal MTV remained as a significant prognostic factor for OS, but nodal MTV did not (HR 1.072, 95%CI 1.019–1.129, *P* = 0.008 for extranodal MTV; HR 1.039, 95%CI 0.951–1.135, *P* = 0.396 for nodal MTV). Three pairs of contrasting cases are presented in supplementary Fig. 2, each pair of two cases that had opposite outcomes due to high and low extranodal MTV despite having equal total MTV.

**Table 2:** Multivariate Cox proportional hazards model for all-cause mortality using TMTV as a covariate

	HR (95% CI)	*P* value
Male sex	1.257 (0.752–2.100)	0.383
Age > 60 years	1.263 (0.567–2.813)	0.568
ECOG PS ≥ 2	3.391 (1.948–5.904)	< 0.001
Stage ≥ 3	1.243 (0.667–2.318)	0.494
CCI score (/point)	1.086 (0.926–1.274)	0.309
TMTV (/100 cm^3^)	1.059 (1.007–1.113)	0.025

Of the MTV indices, TMTV is used as a covariate in this model

*CCI*, Charlson Comorbidity Index; *CI*, confidence interval; *ECOG PS*, Eastern Cooperative Oncology Group performance status; *HR*, hazards ratio; *TMTV*, total metabolic tumor volume

**Table 3:** Multivariate Cox proportional hazards model for all-cause mortality using MTV of nodal and extranodal sites as covariates

	HR (95% CI)	*P* value
Male sex	1.244 (0.744–2.079)	0.406
Age > 60 years	1.263 (0.568–2.810)	0.567
ECOG PS ≥ 2	3.470 (1.979–6.084)	< 0.001
Stage ≥ 3	1.255 (0.676–2.332)	0.472
CCI score (/point)	1.091 (0.928–1.282)	0.291
MTV of nodal sites (/100 cm^3^)	1.039 (0.951–1.135)	0.396
MTV of extranodal sites (/100 cm^3^)	1.072 (1.019–1.129)	0.008

Of the MTV indices, both the MTV of nodal and extranodal sites were used as covariates in this model

*CCI*, Charlson Comorbidity Index; *CI*, confidence interval; *ECOG PS*, Eastern Cooperative Oncology Group performance status; *HR*, hazards ratio; *MTV*, metabolic tumor volume

**Fig. 1:**
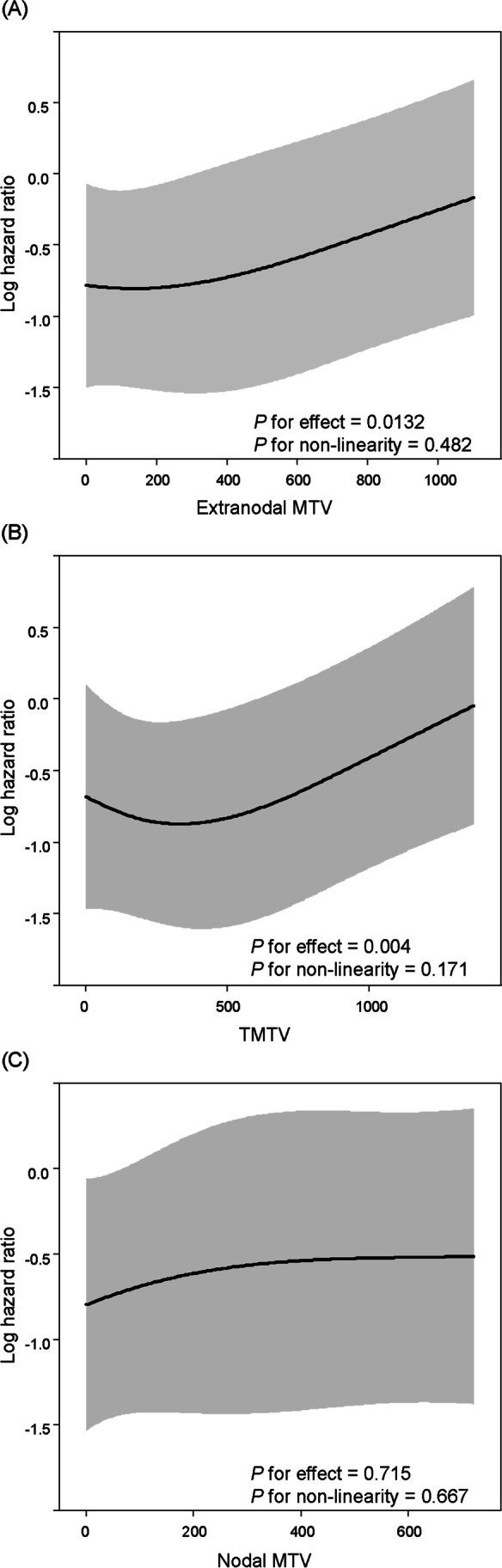
Association between each of the extranodal MTV (**A**), TMTV (**B**), and nodal MTV (**C**), and all-cause mortality risk using a multivariate Cox hazards model with restricted cubic spline with 3 knots. The solid line represents the log hazard ratio, and the shaded area is the 95% confidence interval. MTV = metabolic tumor volume, TMTV = total metabolic tumor volume

To visualize the relationship between MTV and mortality risk in the real world, multivariate Cox hazard modelling with RCS was performed (Fig. 1). A linear relationship with OS was observed for each of the extranodal MTV and total MTV (*P* for non-linearity = 0.482, *P* for effect of extranodal MTV = 0.0132, *P* for non-linearity = 0.171, *P* for effect of total MTV = 0.004). A gradual increase in risk of mortality as MTV increased was observed in the graphs of both extranodal MTV and total MTV. Visually, the graphs of extranodal MTV and total MTV showed clear similarity. Nodal MTV, on the other hand, displayed a completely different shape from extranodal and total MTV graphs, indicating that nodal MTV does not influence the risk of mortality (*P* for non-linearity = 0.667, *P* for effect of nodal MTV = 0.715).

### Prognostic impact of extranodal involvements for OS

A Cox proportional hazard model was developed to evaluate the prognostic impact of differences in the methods of assessing extranodal lesions (Table 4). Both numbers of extranodal involvements and extranodal MTV were used as covariables in this model. Multivariate analysis identified extranodal MTV as a significant prognosticator of OS, while the number of extranodal lesions was not (HR 1.070, 95%CI 1.017–1.127, *P* = 0.009 for extranodal MTV; HR 0.844, 95%CI 0.463–1.540, *P* = 0.581 for number of extranodal involvements).

**Table 4:** Multivariate Cox proportional hazards model for all-cause mortality using the number of extranodal sites and MTV of extranodal sites as covariates

	HR (95% CI)	*P* value
Male sex	1.215 (0.728–2.026)	0.456
Age > 60 years	1.235 (0.555–2.752)	0.605
ECOG PS ≥ 2	3.473 (1.967–6.132)	< 0.001
Stage ≥ 3	1.468 (0.750–2.871)	0.263
CCI score (/point)	1.086 (0.922–1.278)	0.324
Number of extranodal sites ≥ 2	0.844 (0.463–1.540)	0.581
MTV of extranodal sites (/100 cm^3^)	1.070 (1.017–1.127)	0.009

Of the indices assessing the extranodal sites, both the number of extranodal sites and the MTV of extranodal sites were used as covariates in this model

*CCI*, Charlson Comorbidity Index; *CI*, confidence interval; *ECOG PS*, Eastern Cooperative Oncology Group performance status; *HR*, hazards ratio; *MTV*, metabolic tumor volume

**Fig. 2:**
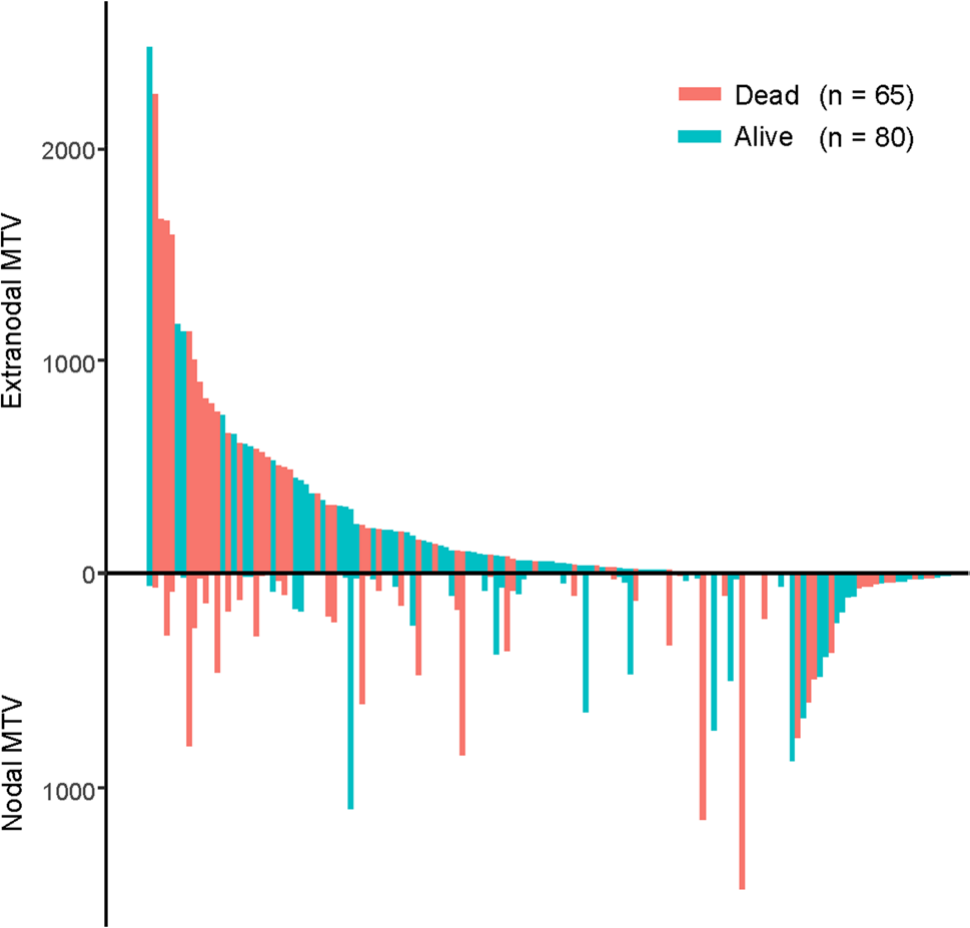
Plots of extranodal MTV by patients. MTV = metabolic tumor volume

Waterfall plots in order of increasing extranodal MTV were created to assess the distribution of extranodal and nodal MTVs in all patients. Patients with high extranodal MTV tend to have lethal outcomes, regardless of whether nodal MTV is high or low (Fig. 2).

## Discussion

The present study showed that the MTV of extranodal involvement measured by FDG PET has an independent effect on survival in de novo DLBCL. We found that the adverse impact of the MTV of total lymphoma lesions on the prognosis seems to depend on the effect of the MTV on extranodal lesions. Compared to MTV of extranodal lesions, MTV of nodal lesions did not affect the prognosis of DLBCL. Furthermore, the prognostic impact of extranodal lesions was greater for MTV than for the number of them.

The MTV of extranodal involvement has a potentially more powerful prognostic impact than the MTV of nodal involvement. As previously reported [16–18], total MTV had an impact on prognosis in our study. However, surprisingly, nodal MTV was not extracted as a significant prognostic factor by multivariate Cox modelling. The known prognostic impact of total MTV on prognosis is dependent on extranodal MTV, as visually demonstrated by the RCS-Cox model. Significant molecular differences have been noted between nodal and extranodal DLBCL [8–10], leading to a negative impact of extranodal involvement on prognosis [6, 7]. Bone marrow is one of the most frequently involved extranodal organs and is also an independent poor prognostic factor for DLBCL [6]. Patients with bone marrow involvement had a higher LDH level and tumor burden [29]. Tumor burden also had an adverse impact on prognosis in DLBCL [11–13]. In our study, approximately 20% of the extranodal MTV group had bone marrow involvement, higher than the previous report [6], which might have led to a high tumor burden and poor prognosis in the present study.

The prognostic impact of extranodal involvement has been noted and included in the IPI, the most widely used prognostic model [30]. In that model, not the volume, but rather the number of extranodal sites is used to assess the impact on prognosis. We found that the prognostic impact of extranodal involvement depends predominantly on the MTV, rather than the number of lesions. The prognostic impact of extranodal involvement may differ depending on the organ [6]. Organ-specific extranodal MTV validation will pose a challenge for personalised medicine in the future. MTV as measured by FDG-PET is an established indicator of tumor burden and metabolism, reflecting the aggressiveness of lymphoma [16–18]. Since FDG-PET offers the highest accuracy for detecting extranodal lymphoma [14, 15], measuring extranodal MTV might facilitate individualised treatment strategies.

Most of the previous studies have evaluated the prognostic impact of MTV calculated as total MTV, which includes both nodal and extranodal involvements. A previous study concluded that the nodal MTV had more predictive power than Ann Arbor stage [31]. In this previous report, patients with stages I and IV were excluded, as stage II or III can change the IPI score. Since stages I and IV are excluded, the total MTV in this study is automatically equal to the nodal MTV. As in this previous report, few reports have evaluated and directly compared MTV separately for nodal and extranodal involvements. The results of our study provide a reasonable answer to fill up the evidence gap on whether nodal or extranodal involvement plays a more important role in the prognostic impact of total MTV.

Our study has some limitations. First, the results of the present study need to be interpreted by taking reporting bias into account, given the study. Second, this was a single-centre study with moderate sample size, placing certain limitations on the statistical power of this analysis. However, no previous studies have focused on the MTV of extranodal lesions, and our study provides new insights into the understanding of the MTV as a prognostic factor for DLBCL. Third, we should note that patients with very poor activities of daily living or aggressive progression of lymphoma such that PET cannot be taken were not included. Patients requiring assistance during imaging are not candidates for receiving FDG-PET, to avoid radiation exposure to radiographers. In Japan, inpatient PET scans are not covered by universal insurance. Patients with aggressive presentation of the lymphoma, such as those admitted to the hospital in an emergency, are therefore not eligible for PET. Some degree of selection bias was thus unavoidable in the present study. Finally, the observed proportion of patients with extranodal involvements in the present study was 79.3%, a bit higher than what would be expected [30, 32]. We attribute this difference to the fact that our study population was older than those of the previous reports. Aging has been known to increase the frequency of extranodal involvements in DLBCL [7]. Reflecting on the recent world aging, understanding and managing patients with extranodal involvement in DLBCL will become more important in the future.

In conclusion, the significant prognostic impact of the MTV of extranodal involvement measured by FDG PET was demonstrated in patients with de novo DLBCL. Among total MTV of DLBCL lesions, extranodal MTV potentially had a stronger prognostic impact than nodal MTV. Regarding the prognostic influence of the extranodal sites, MTV is more important than the number of extranodal sites, as used in the IPI.

## Supplementary Information

Below is the link to the electronic supplementary material.

Supplementary figure 1. Flow chart of patient selection. CNS = central nervous system; DLBCL = diffuse large B-cell lymphoma; FDG-PET = ^18^F-fluorodeoxyglucose positron emission tomography. (PNG 17 kb)

High resolution image (TIFF 14 kb)

Supplementary figure 2. FDG-PET images of patients of early death group and long-term survival group, respectively. Group A has a high nodal MTV but a good prognosis (survived more than 2 years). Group B is the patients whose total MTV is comparable to that of Group A, the group directly above each case, but whose extranodal MTV is high and the prognosis is poor (death within 1 year). FDG-PET = ^18^F-fluorodeoxyglucose positron emission tomography, MTV = metabolic tumor volume. (PNG 678 kb)

High resolution image (TIF 16504 kb)

## Data Availability

Data sharing not applicable to this article as no datasets were generated or analysed during the current study.
