# Supplementary figures and images for: Prognostic value of metabolic tumor volume of extranodal involvement in diffuse large B cell lymphoma
Source: Ann Hematol. 2023 Mar 23;102(5):1141–8. doi: 10.1007/s00277-023-05165-x (PMC10102098; doi:10.1007/s00277-023-05165-x)

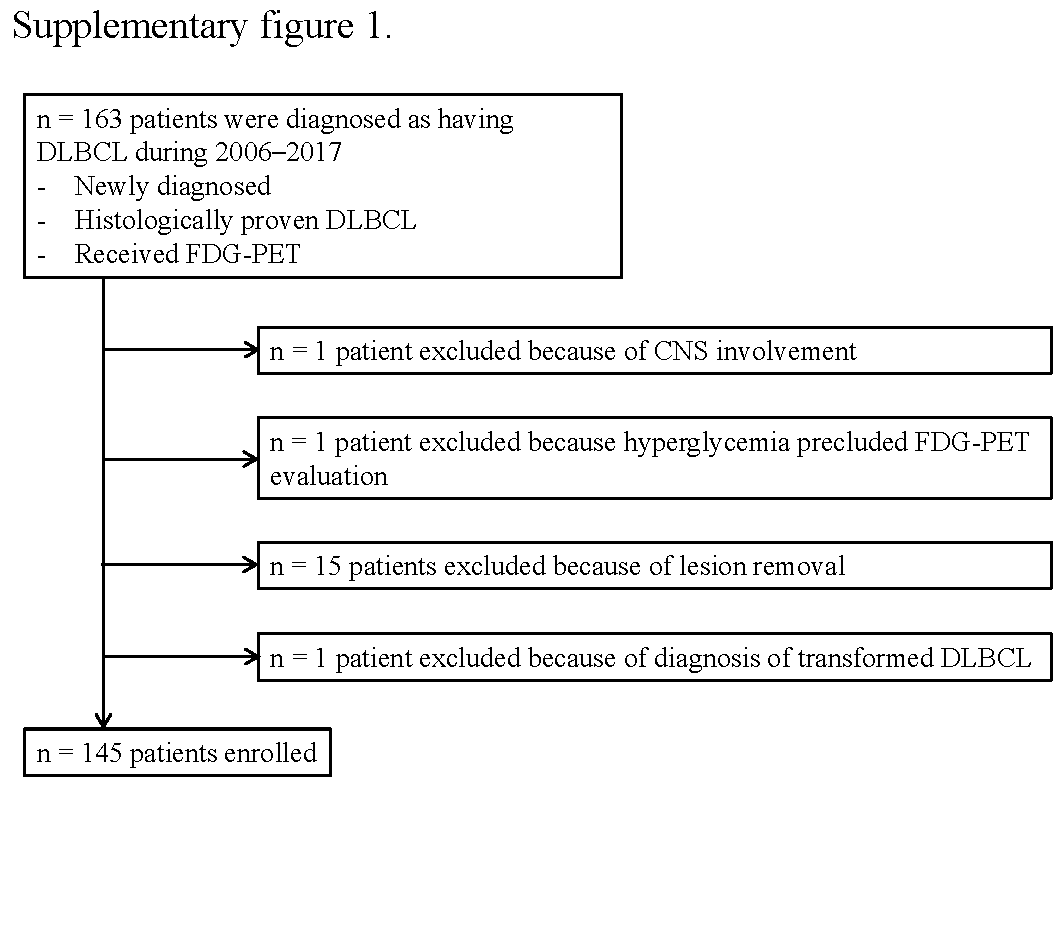

Supplement: Supplementary file 1 — Flow chart of patient selection. CNS = central nervous system; DLBCL = diffuse large B-cell lymphoma; FDG-PET = 18F-fluorodeoxyglucose positron emission tomography. (PNG 17 kb) [file 277_2023_5165_Fig3_ESM.png]

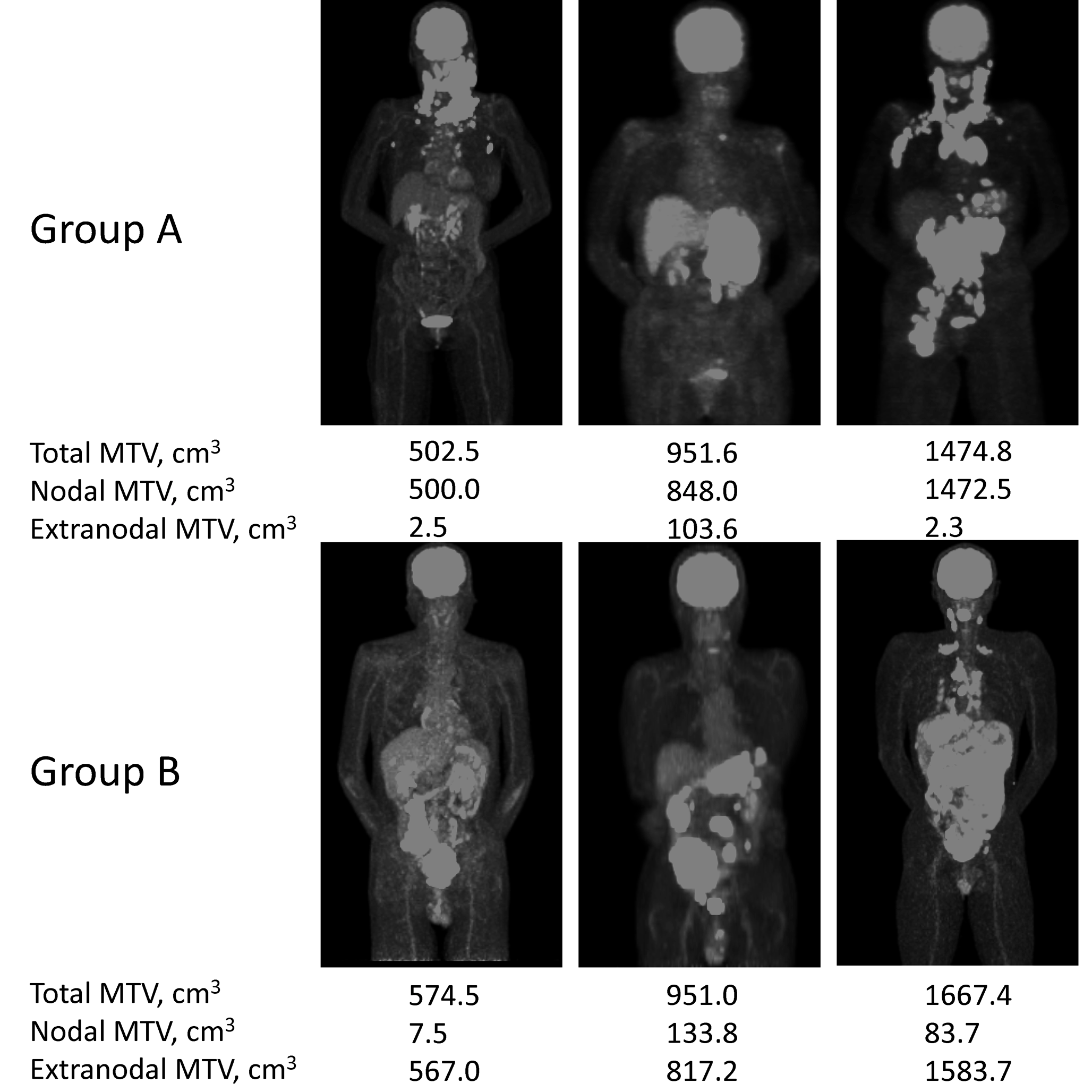

Supplement: Supplementary file 3 — FDG-PET images of patients of early death group and long-term survival group, respectively. Group A has a high nodal MTV but a good prognosis (survived more than 2 years). Group B is the patients whose total MTV is comparable to that of Group A, the group directly above each case, but whose extranodal MTV is high and the prognosis is poor (death within 1 year). FDG-PET = 18F-fluorodeoxyglucose positron emission tomography, MTV = metabolic tumor volume. (PNG 678 kb) [file 277_2023_5165_Fig4_ESM.png]

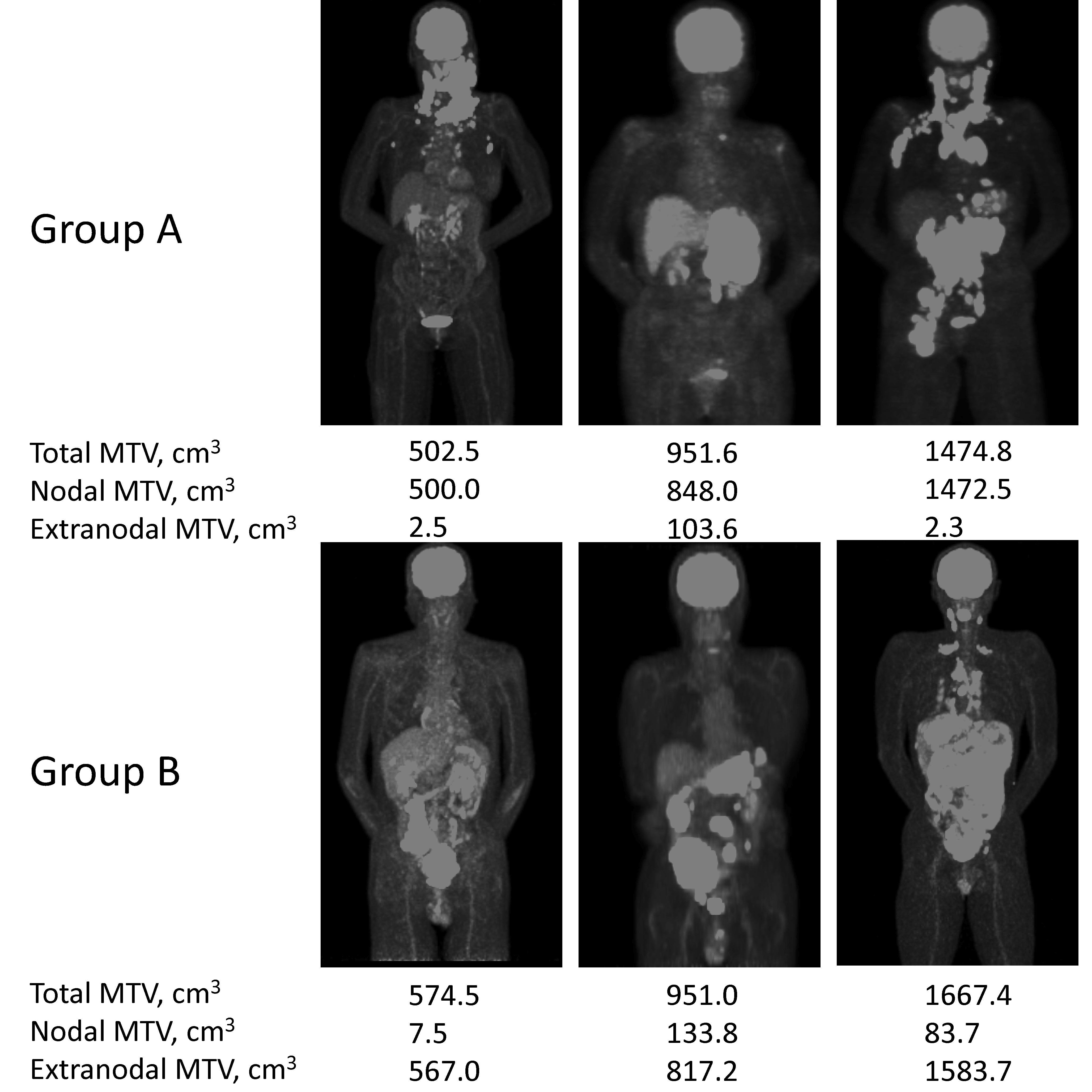

Supplement: Supplementary file 4 — High resolution image (TIF 16504 kb) [file 277_2023_5165_MOESM2_ESM.tif]
